# Application of static and impulse magnetic fields to bacteria *Rhodospirillum rubrum* VKM B-1621

**DOI:** 10.1186/s13568-017-0362-9

**Published:** 2017-03-11

**Authors:** Galina Khokhlova, Mikhail Vainshtein

**Affiliations:** 1 Institute of Biochemistry and Physiology of Microorganisms, Russian Academy of Sciences, Prospekt Nauki 5, Pushchino, Moscow region 142290 Russia; 2 Pushchino State Institute of Natural Sciences, Prospekt Nauki 3, Pushchino, Moscow region 142290 Russia

**Keywords:** *Rhodospirillum rubrum*, Magnetic fields, Amylase activity

## Abstract

The paper presents effects of different magnetic fields (MFs) (static—SMF and impulse—IMF) on bacteria *Rhodospirillum rubrum* VKM B-1621. The MFs had different magnetic strength: SMF—up to 173 mT; IMF—25 mT. The studied object was amylase activity which was measured by decrease in the starch concentration during incubation in the MFs. The term of incubation in the MFs was limited with 2 h. The SMF affected neither amylase activity of *R. rubrum* nor standard deviation in distribution of the residual starch concentration along the plate but the IMF did. The IMS effects varied along the plate which could be related with distance from the magnetic center of the applied device. In whole, application of impulse MFs can regulate bacterial activity and thus could be used for biotechnological application.

## Introduction

Usually, microbiological and biotechnological investigations include study on effects of different abiotic agents on bacterial activities. Despite of increasing stock of these data, there is an area that remains unclear, namely: publications about effects of magnetic fields on bacteria contain contradictory data or not comparable. Drastic difference in the discovered results might be explained by different investigated objects, different type and power of applied magnetic fields, etc.

Meanwhile, reported effects of magnetic fields on bacteria present an interest not only for basic and general microbiology but, as well, for biotechnological purposes: the publications declared that magnetic fields could change or regulate bacterial activity. The most attractive area of application of possible magnetic effects is medicine: (1) bacteria are the most simple model of living organism for investigation of magnetic effects, (2) some bacteria play a significant role as infection agents, pathogens, or, in contrast, as beneficial symbionts of the human microbiota. According to the last reason, modern scientific investigations focused mainly on inhibition of pathogens with magnetic fields for medical purposes. For example, some investigations showed that 1-day application of the static magnet field (60 mT) reduced number of dental plaque microbes in vitro (Brkovic et al. 2015). Some other investigators showed decrease in the number of colony forming units (CFU) produced by application of altering electromagnetic fields (Ahmed et al. 2013; Bayır et al. 2015). The most drastic decrease in CFU (at least 20%) was achieved with the exposure at 300 Hz and 1.5 mT. In contrast to these data, application of the altering electromagnetic field (2 mT, 50 Hz) or static magnetic field (200 mT) to some pathogenic bacteria discovered just a slight decrease in the bacteria number only after the first hours of incubation and the following increase in 24  h (Mihoub et al. 2012; Segatore et al. 2012). As well, rotating altering magnetic field (1–50 Hz; 22–34 mT; 1 h) increased growth, metabolic activity, and share of bacterial cells in biofilms produced by *Staphylococcus aureus* and *Escherichia coli* (Fijalkowski et al. 2013).

Thus, the available data on the influence of magnetic fields are contradictory. We can suggest that the main problems hindering any investigation on bacterial response to applied magnetic fields are:

- Fluctuations of geomagnetic field as some changes in magnetic background during long-drawn-out experiments which mean necessity of the short-term experiments;
- Possible different responses of different intracellular magnetic recipients during long exhibition, consequently, lead to an uncertain outcome response of the cell as a whole, i.e. it is interesting to measure activities separately;
- Possible different bacterial responses at different stages of growth which could be measured, at least, at different levels of the substrate utilization.

Our paper presents results of short-term experiments on effects of different magnetic fields (static magnetic field; impulse magnetic field of physiotherapeutic device) on amylase activity of bacteria *Rhodospirillum rubrum* VKM B-1621. The choice of starch as substrate is based on our earlier experiments (Anisimov et al. 2005) where different effects of magnetic field were shown for ^14^C-labelled carbonate penetrating into cells through membrane. This time, in contrast, we studied possible magnetic effects on utilization of polymer organic substrate which could be less affected by membrane charge. The main tasks were to analyze (a) if different types of magnetic fields can affect the same type of bacterial activity, (b) if the effects were the same at different levels of growth measured as different levels of the residual substrate.

## Materials and methods

### Microorganisms and nutrient media

The studied bacterial strain was *R. rubrum* VKM B-1621 deposed in the All-Russian Collection of Microorganisms (VKM). The culture is presented with classical Gram-negative purple photosynthetic bacteria. The species was chosen by its ability to different types of growth: (1) photoheterotrophic under illumination (Ivanovsky et al. 1997), (2) chemoheterotrophic with nitrate as electron acceptor (Katoh 1963), (3) organoheterotrophic with atmospheric oxygen as oxidant (Oelze and Weaver 1982) or with anaerobic fermentation (Schön and Biedermann 1972; Schultz and Weaver 1982). It was shown also that some purple bacteria were affected by magnetic field during phototrophic growth (Haberkorn and Michel-Beyerle 1979) and some of them can form magnet-sensitive intracellular inclusions (Vainshtein et al. 1997). As well, it was shown that this strain *R. rubrum* VKM B-1621 is capable of forming intracellular magnet-sensitive cobalt- or chromium-containing inclusions (Ariskina et al. 2004).

Purity of the culture was checked both by phase contrast light microscopy and by inoculation of the solidified rich nutrient medium “5/5 IBPM” (the Russian nutrient medium similar with the tryptic soy agar) to seek possible contaminants.

To pre-grow bacteria for the experiments, the modified medium DSMZ 27 was prepared (https://www.dsmz.de/microorganisms/medium/pdf/DSMZ_Medium27.pdf) (g/l): yeast extract—0.30, l-lactate—0.49, (NH_4_)-acetate—0.50, Fe(III) citrate solution (0.1% in H_2_O)—5.00 ml, KH_2_PO_4_—0.50, MgSO_4_ × 7 H_2_O—0.40, NaNO_3_—0.34, NH_4_Cl—0.40, CaCl_2_ × 2 H_2_O—0.05, trace element solution SL-6—1.00 ml. pH was adjusted to 6.8–7.0 with NaOH (2%). In this medium, bacteria were grown in hermetically closed Balch vials under anaerobic conditions at ~2 kl ×(tungsten lamp) and at ~25 °C till appearance of visually perceptible red suspension (equal approx. 10^7^ cells/ml).

For experiments on amylase activity, the pre-grown bacterial suspension was placed into the modified medium with potato starch (2.0 g/l), pH 6.8–7.0, and then transferred into wells of the 96 well plate (A–H × 1–12), 40 μl per well.

### Application of static magnetic field

**Fig. 1:**
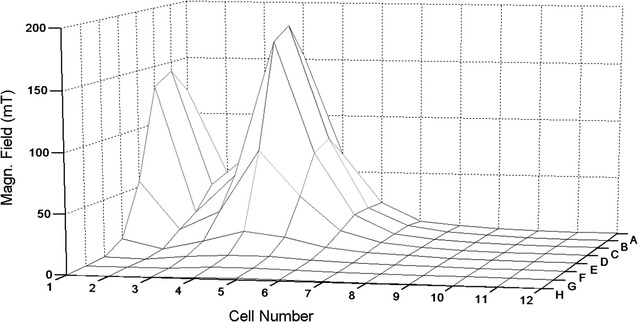
Distribution of the static magnet field (SMF) in 96 wells of the plate. *Axis Z* strength of the SMF, mT; *X rows* of the wells, 1–12; *Y columns* of the wells, *A–H*

In experiments with static magnetic field (SMF), the culture was placed into 96 wells of the well plate (A–H × 1–12), 40 μl per well, and exposed with applied metal magnet. Position of the metal magnet was same in all experiments with the SMF: it was placed under the well plate with location A–C × 1–3. Strength of the applied SMF was measured in each well of the plate with the magnetometer ATE-8702 (Aktakom, Russia). Distribution and strength of the SMF in the plate is shown in Fig. 1. The measured maximum values exceeded 150 mT, their distribution on the plate was patchy and thus provided possibility to find zones of magnets effects on bacteria if any effects present. The treatment lasted 3 h.

### Application of impulse magnetic field

Physiotherapeutic device “PhotoSPOK” (Magnomed, Belorussia) was used to form impulse magnetic field (IMF). Description of the device is presented both at the company site http://www.magnomed.by/glavnaya/nasha-produktsiya/fotospok (in Russian) and in the patent (Pletnev et al. 2010). Published technical characteristics of this equipment are limited with the following information: pulse packet repetition rate is up to 10 Hz, signal frequency in pulse packet is up to 200 Hz, the magnetic field is emitted by pulses with a frequency of 1200 pulses/s within preset time intervals (Pletnev et al. 2010). Beside any other information which is a commercial “know how” of the company, the mentioned one was necessary and sufficient for our purposes, namely: to know that we experimented with a pulsed magnetic field of 25 mT.

The device was placed on the cover of the 96-well plate (A–H × 1–12), the device center was located over the columns 5 and 6 of the well plate. The device includes a small luminous screen, thus, to avoid any lighting effects on bacteria, we closed the screen with two layers of aluminum foil. Each well contained 40 μl of the bacterial suspension. The magnetic treatment lasted 2 h. To avoid any heating during the device work, every 10 min the device was turned off for the next 10 min. Additional experiments with a thermoisolating gasket (15 sheets of paper) discovered no heating effects in the experiments.

### Analyses of amylase activity

Effects of magnetic fields on amylase activity were measured by comparison of the residual concentration of starch in the medium (Xiao et al. 2006). The starch concentration was determined as optical density (OD) at 595 nm after reaction of starch with iodine solution; our modification of the iodine solution was (mg/l): iodine—8.9, potassium iodide—17.8, glycerol—840.3, acetic acid—737.5. The described supplementation of the modified solution with acetic acid was essential to stop biochemical transformation and to provide stable quantitative reaction of iodine.

To stop biological processes and to form the color reaction, 40 μl sample in each well of the 96 well plate was supplemented with 60 μl iodine solution. The maximal initial starch concentration (100%) was equal to optical density (OD) 3.38. Measurements were carried out with microplate reader (Bio-Rad, iMark) at 595 nm in the 96 well plate (well columns A–H, well rows 1–12).

### Statistics

Statistical analyses were provided with standard methods, including treatment with the Excel statistics. Analysis of variance (ANOVA) for the single factor was used to determine if the means of different groups of the data were equal. The statistics in the text below are: arithmetic mean (x), standard deviation (s), sample variance (s^2^), and the degrees of freedom (DF).

## Results

There are publications which witness effects of weak altering magnetic fields on glycosidase activity in higher organisms where authors defined glycosidase activity as maltase and amylolytic activities measured separately (Filippov et al. 2014; Kuzmina et al. 2015). They discovered some effects of the applied magnetic fields, for example—artificial geomagnetic storms—on amylase activity. As well, their colleagues reported that in the artificial magnetic fields both amylolytic activity and Michaelis constant of the starch hydrolysis in the intestine of fish were higher than in the control group (Golovanova et al. 2016). Yet, it was unknown if amylase activity was affected by the applied magnetic fields directly, as a primary target, or indirectly.

### Amylase activity in geomagnetic field (blank)

Bacterial growth was accompanied by a diminution of the starch in the medium by optical density. Samples from the flask into the well plate were taken 4 times during active growth; these different stages presented different levels of the substrate concentrations, namely: 98, 79, 68, and 60% of its initial concentration. In geomagnetic field distribution of residual starch in the wells of the 96 well plate was uniform and smooth along the plate: the average mean of the optical density for residual starch changed from 3.26 to 1.98 while the standard deviation of the OD along 12 columns of wells in the plate varied just in limits 0.006–0.009 (Table 1).

**Table 1:** Statistics: residual concentrations of starch (measured by optical density after reaction with iodine) in the *R. rubrum* culture after 2 h exposition in the geomagnetic field (Blank) and in the impulse magnet field (IMF)

Level of growth as starch concentration by OD, % of initial	98	79	68	60
Geomagnetic field (blank)				
Final starch concentration in the experiment, OD, average mean (x)	3.26	2.63	2.24	1.98
OD standard deviation (s) for 12 columns	0.006	0.009	0.007	0.007
Impulse magnet field (IMF)				
Final starch concentration in the experiment, OD, average mean (x)	3.18	2.01	1.84	1.92
OD standard deviation (s) for 12 columns	0.021	0.200	0.050	0.037

Number of the data in each group was presented by 96 wells of plate (8 rows, 12 columns)

### Amylase activity in static magnetic field (SMF)

The scheme of distribution of the SMF on the 96 well plate is presented in Fig. 1. To analyze results of the experiment, the area was divided arbitrarily into the following ranges (groups) according to strength of the applied magnetic field: 0–0.5, 0.6–1.0, 1.1–3.0, 3.0–16.0, 16.0–85, and 120–173 mT. Statistic results are shown in the Table 2: average means of the residual starch concentrations were equal in all magnetic bands and the OD arithmetic average mean (x) for the full amount of the data was 3.20 while the OD standard deviation (s) was 0.041. There were no SMF effects on the *R. rubrum* amylase activity after 2 h of the exposition: comparison of the data at 0.0–0.5 mT and other data by the ANOVA analysis showed that there is no difference between the groups of the data (p < 95%).

**Table 2:** Statistics: residual concentrations of starch (measured by optical density after reaction with iodine) in the *R. rubrum* culture after 2 h exposition in the static magnet field (SMF)

Nos. of the data groups	Ranges of the SMF, mT	Number of wells (number of the data in the group, n)	OD	
			Average mean (x)	Sample variance (s^2^)
1	0.0–0.5	18	3.01	0.285256
2	0.6–1.0	18	3.27	0.103128
3	1.1–3.0	18	3.25	0.252374
4	3.6–16.0	18	3.27	0.144751
5	16.0–85	18	3.22	0.165431
6	120–173	6	3.15	0.228619
${\text{s}}_{\text{A}}^{2}$	0.182701			
${\text{s}}_{\text{G}}^{2}$	0.116315			
F = ${\text{s}}_{\text{A}}^{2} /{\text{s}}_{\text{G}}^{2}$	1.57075			
F_0.95_	2.315689			

F < F_0.95_ thus, there is no difference between the groups of the data at the 95% level of degrees of freedom

### Amylase activity in alternating impulse magnetic field (IMF)

**Fig. 2:**
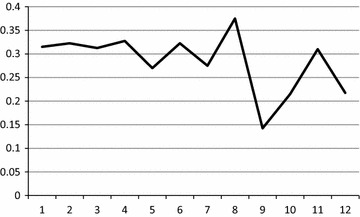
Effects of the applied impulse magnetic field on amylase activity of *R. rubrum* presented as difference between residual starch concentrations in IMF and in geomagnetic field in wells along 12 columns (*axis X*) of the 96 well plate after exhibition, OD average (4 experiments × 8* rows* for each* column*)

In contrast to the smooth distribution in SMF and in geomagnetic field, distribution of residual starch showed extremely uneven distribution in the IMF. This is evident from the data presented in the Table 1: OD standard deviation (s) for 12 columns varied from 0.05 to 0.20. As well, application of the device resulted in a deviation from blank which was distributed along the plate irregularly (Fig. 2). In total, we can suggest that IMF affected amylase activity, directly or indirectly. We have no enough information about affecting mechanisms, so, the topic is open for discussion.

## Discussion

Study on effects of magnetic fields on bacteria is a very interesting subject for microbiology and biotechnology. Till present, possible targets and mechanisms of these effects in bacterial cells are unclear; the published data were explained with distinct hypotheses and theories. It is very hard to compare the published results because they are received with different species, with different types of metabolism, and with different types of the cellular wall. Anyway, investigations of effects of magnetic fields are essential for new knowledge and possible regulation of bacterial activity.

The cited data suggest some effects of magnetic fields, but do not provide exact information about type of response. It has already been shown that the responses or effects of magnetic fields could be different at different stages of bacterial growth (Anisimov et al. 2005; Segatore et al. 2012). We believe the study at different stages of growth is important. Our methodical approach with application of the 96 wells plate was handy to study the short-term impact at the different levels of growth measured by residual substrate (98, 79, 68, and 60% of initial concentration).

In total, our experiments on effects of magnetic fields on *R. rubrum* resulted in the following new data:

i. Application of the strong static magnetic field (SMF, up to 173 mT) did not affect amylase activity during 3 h exposition according to verification by analysis of variance (ANOVA);
ii. Application of the impulse magnetic field (IMF, 25 mT) affected amylase activity during 2 h exposition which was verified both by increase of the standard deviation (s) for 12 columns of the plate (Table 1: from 0.006–0.009 to 0.021–0.200) and by irregularity of the starch utilization along the plate (Fig. 2).

Different effects of MFs on bacterial growth need some hypothesis to explain them.

One of the possible hypotheses could be based on change in permeability of charged membranes of bacterial cells. In our preliminary studies with artificial “geomagnetic storm”, we suggested different introduction of dissolved iron into cells under different magnetic conditions (Anisimov et al. 2005). A test on increase of permeability was performed with propidium iodide and uptake of silica nanospheres for 4 cocci of different taxa (Nguyen et al. 2015) and the results showed that exposing the bacteria to an electromagnetic field (EMF, exposition at the microwave frequency of 18 GHz) induced permeability in the bacterial membranes, the cells remained permeable for at least 9 min after the EMF exposure. Anyway, this approach is shown for alternating MFs but not for SMF.

Another old hypothesis is for the weak alternating magnetic fields and suggests a resonance frequency interaction of these weak MFs with biological systems. Numerous enzymes contain a key inorganic ion, for example, Ca^2+^-binding protein (amylase) could be a charged oscillator (Lednev 1991). This approach was proposed for altering MFs but not for SMF. There is a possibility too that bacterial cells can contain different enzymes with different responses (including opposition reactions) to the same MF application.

There is also a hypothesis that SMF can modify the secondary structure of *E. coli* proteins. Potenza et al. (2004) reported that SMF with strength 300 mT induced cell proliferation and changes in the *E. coli* genes expression.

Our data did not allow to find a final unique solution of these hypotheses, but showed that used methodical approach permits to discover effects of MFs on bacterial activity in short-term experiments. In whole, application of magnetic field can regulate bacterial activity and thus has a significant biotechnological potential. Alternating magnetic field showed more effects than the static one.
